# An Audit of Factors Impacting the Time to Resolution of the Metabolic Parameters in Diabetic Ketoacidosis Patients

**DOI:** 10.7759/cureus.31142

**Published:** 2022-11-06

**Authors:** Muath F AlWahbi, Sami H Alharbi, Saleh A Almesned, Faisal A Alfawzan, Rayan T Alsager, Abdullah A AlHojailan, Emad A Alfadhel, Fahad G Al-Harbi

**Affiliations:** 1 Diabetes and Endocrinology, King Fahad Specialised Hospital, Buraydah, SAU; 2 Internal Medicine and Pulmonologist, King Fahad Specialised Hospital, Buraydah, SAU; 3 Medicaine, Qassim University, Buraydah, SAU; 4 Medicine, Qassim University, Buraydah, SAU

**Keywords:** saudi arabia, retrospective, metabolic parameters, ketoacidosis, diabetes

## Abstract

Introduction: Diabetic ketoacidosis (DKA) is a major complication affecting patients with diabetes. It is often the first presentation of type I diabetes and can also occur due to a lack of compliance with insulin therapy or infection, among other causes. Hospitalizations for DKA have increased globally among patients with type I and type II diabetes, which poses a strain on health systems.

Aim: To determine the factors impacting the time to resolution of metabolic parameters in DKA patients.

Methods: This retrospective study was performed by reviewing the clinical records of hospitalized DKA patients at King Fahad Specialist Hospital, a tertiary-level healthcare center in Buraidah, Al Qassim region, Saudi Arabia. The study included all DKA admissions that met the inclusion criteria between September 2019 and April 2022.

Results: A total of 129 patients with a diagnosis of DKA from January 2019 to April 2022 were identified. Of these, 67 patients met the inclusion criteria. More females experienced DKA (56.7%), and the study population had an improvement rate of 97%. The mean length of stay (LOS) for patients with DKA was 73.19 hours, and the median time for DKA resolution was 15 hours (mean time 21.38 hours). The omission of insulin was the leading precipitating factor for DKA (43%) followed by infection (12%). A high serum bicarbonate (HCO_3)_ level was identified as an independent predictor for a longer time to resolution. Patients with DKA who have high glucose levels on admission, higher body mass index (BMI), older age, and higher weight stayed in the hospital for extended periods. Conversely, patients with a higher serum potential of Hydrogen (pH) had a shorter LOS. Age was the only clinically independent predictor for a minimum LOS for DKA. Patients with comorbidities had a longer hospital LOS than patients without comorbidities; no such relationship has been reported in other studies. Patients in our study population had a longer time of resolution than observed in other studies, although no clear cause was identified.

Conclusion: This study contributes to our understanding of DKA in hospitalized patients in Saudi Arabia. This is the first study to link patients with DKA who have comorbidities to a longer hospital stay in the kingdom of Saudi Arabia. This study also identified multiple clinical and biochemical parameters related to the variability in LOS and time to resolution of DKA in hospitalized patients

## Introduction

Diabetic ketoacidosis (DKA) is a significant condition that includes the biochemical features of hyperglycemia, acidosis, and ketonemia [1]. It occurs as a complication in diabetic patients and leads to a buildup of ketone bodies, which can lead to life-threatening conditions [2].

The incidence of patients with type 1 diabetes presenting with DKA in Arab and African countries ranges from 12% to 17% [3,4]. Saudi Arabia has the highest number of type 1 diabetes patients (36.3%) among Arab countries, with a DKA incidence of 25% to 80%. Overall, in Arab countries, the rate of DKA has been reported as 46.7% among patients with type 1 diabetes. Saudi Arabia and Egypt had the highest rate of DKA, occurring in 60.1% of patients with type 1 diabetes. Kuwait and Saudi Arabia are among the 10 countries with the highest incidence of DKA in the world [5].

Diabetic ketoacidosis is often the first presentation of type I diabetes, but can also occur in patients with diabetes due to many causes. Lack of compliance with insulin therapy is the leading cause of DKA (65%); this is correlated to many psychosocial and socioeconomic factors. Infection represents the second most common cause of DKA (30%) [6]. Other factors include alcoholism, pancreatic disease, stroke, ischemic heart disease, trauma, and medication-induced DKA [7]. In another study, stress was found to account for 10% of DKA cases [8]. Diabetic ketoacidosis is related to morbidity, mortality, and use of health care resources, accounting for 9% to 28% of all diabetes-related hospital admissions [9]. Hospitalizations for DKA have increased globally for both type I and type II diabetes, according to several epidemiological studies [10].

Diabetes and its complications, including DKA, represent a significant cost for countries and governments. A Saudi Arabian study showed that the estimated cost of diabetic patients under treatment was approximately 17 billion Riyals in 2014. The study also predicted a cost of 27 billion Riyals if undiagnosed cases were included. Furthermore, if prediabetic patients progress to become diabetic at the observed rate, the predicted future cost is 43 billion Riyals [11]. The estimated cost of diabetes mellitus in the United Kingdom is 5.5 billion British pounds per year, which includes 3280 British pounds per patient with type I diabetes and 1686 British pounds per patient with type II diabetes [12,13].

To reduce the impact on health systems, it is important to examine hospital length of stay (LOS) and factors that may impact it. A retrospective study at Theptarin Hospital in Thailand showed that the median LOS was three days, and 76.3% of patients were discharged within five days after admission [10]. Another study conducted at St Vincent’s Hospital Melbourne, Australia, revealed that the median LOS was three days (range: two to five days) [14]. Understanding factors that contribute to longer LOS is crucial. One study found that the presence of hyperchloremia in DKA patients delayed the time to resolution and LOS [15]. However, another study found that reduced potential of Hydrogen (pH) and elevated serum potassium levels on admission were independent biochemical markers that increased the time to resolution of DKA [14]. Understanding the precipitating factors of DKA is also essential as they may impact the time to resolution, LOS, and patient outcomes.

This study aims to investigate the factors impacting the time to resolution of metabolic parameters in DKA patients. The results of this study will help predict the LOS in DKA patients according to their biochemical parameters, which may contribute to promoting the management and outcomes of DKA patients. No similar study has been conducted in Qassim or Saudi Arabia.

## Materials and methods

This retrospective study was conducted by reviewing the clinical records of hospitalized DKA patients at the King Fahad Specialist Hospital, a tertiary-level healthcare center in Buraidah, Al Qassim region, Saudi Arabia. All DKA admissions between September 2019 and April 2022 were included in this study.

Patients who met the biochemical criteria for DKA and were 16 years of age or older were included in the study. Based on the American Diabetes Association (ADA) guidelines, DKA was defined as a triad of uncontrolled hyperglycemia, metabolic acidosis, and ketonuria or ketonemia [16]. The parameters included blood glucose > 250 mg/dl, arterial pH < 7.3, bicarbonate ≤ 18 mEq/L, moderate ketonuria or ketonemia, and anion gap >10 mEq/L [15,17]. Euglycemic DKA was defined as DKA without marked hyperglycemia (plasma glucose ≤ 250 mg/dL) in the presence of metabolic acidosis (arterial pH < 7.3, serum bicarbonate < 18 mEq/L) and positive serum ketones [18]. In the case of euglycemic DKA, the history of diabetes was taken into consideration. For each patient, the first episode of DKA within the study period was included [15]. Patients presenting with biochemical evidence of hyperglycemia without ketoacidosis, hyperglycemic hyperosmolar state (HHS) (i.e., serum osmolality >320 mOsm/L), mixed HHS/DKA, or incomplete data were excluded.

The time to resolution of DKA (calculated as the time from the first blood sample that provided a measure of acid-base status to the normalization of key biochemical markers) was the primary measure of this study [14]. Criteria for the resolution of DKA included blood glucose < 200 mg/dL and two of the following criteria: serum bicarbonate (HCO3) level ≥ 15 mEq/L, venous pH > 7.3, and calculated anion gap ≤ 12 mEq/L [16]. The Saudi ministry of health guidelines and protocols for diabetes emergencies are the primary treatment protocol for treating patients [19]. Secondary outcomes included precipitating factors, choice of intravenous fluid, adverse events, and LOS [14]. Variables included age, gender, dates of hospital admission and discharge, diagnosis, and medical comorbidities. Ethical approval was obtained from Qassim Region Research Ethics Committee to conduct this hospital-based research (approval no.: 1433-908871). Participants’ privacy and anonymity were protected throughout the study.

## Results

A total of 129 samples were identified with a diagnosis of DKA from January 2019 to April 2022. A total of 62 patients were excluded for the following reasons: 25 patients were younger than 16 years of age; 27 patients had missing files or data; four patients were found to be out of our study period; six patients had an incorrect diagnosis. In total, 67 patients were included in the analysis and met the following criteria: 16 years of age or older, with an episode occurring between January 2019 and April 2022 meeting the biochemical criteria of DKA.

**Figure 1:**
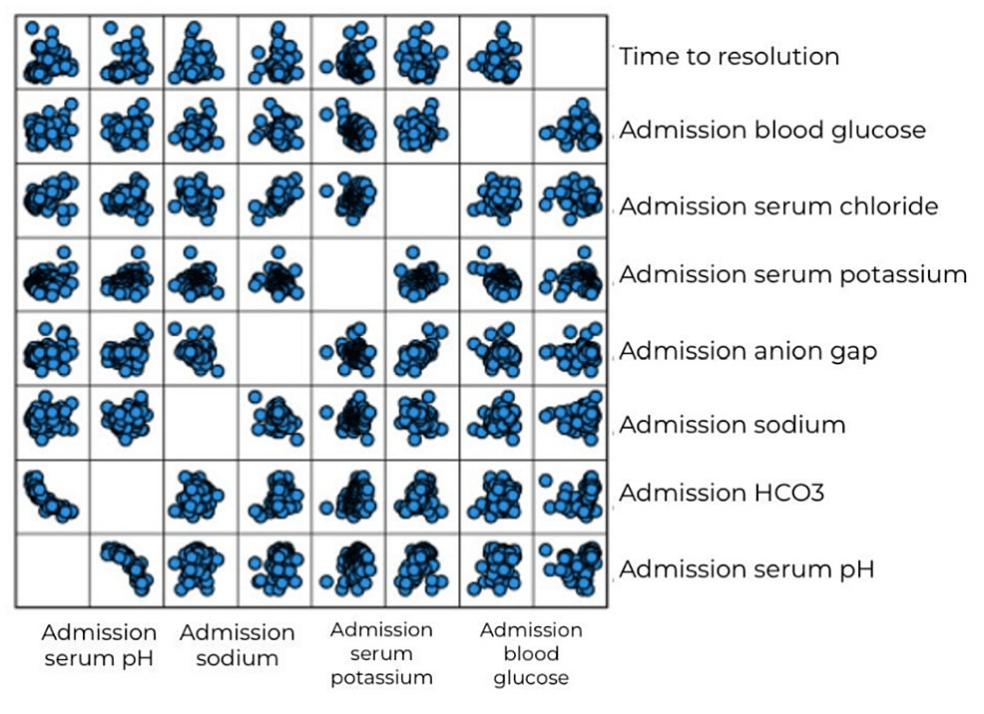
Graphical representation of the linear correlation of time to resolution with metabolic parameters HCO3: Bicarbonate, pH: Potential of Hydrogen

**Figure 2:**
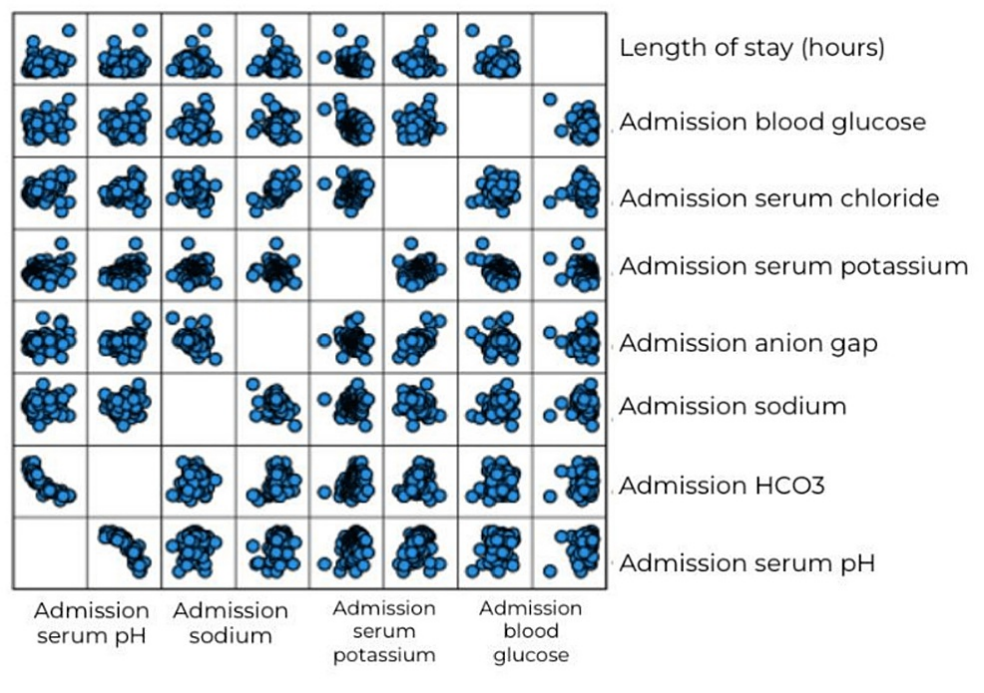
Graphical representation of the linear correlation of hospital length of stay with metabolic parameters. HCO3: Bicarbonate, pH: Potential of Hydrogen

The relationship between variables was linear for most of the major study variables, as is evident from scatter plots (Figures 1, 2). The data were normally distributed as the skewness and kurtosis values were within the range (± 2), except for LOS.

The mean hospital LOS of DKA patients was 73.19 hours. The mean weight, height, and body mass index (BMI) of DKA patients were 61.44 kg, 162.17 cm, and 23.16 kg/m2, respectively. Descriptive statistics of the metabolic parameters of the study population are presented in Table 1.

**Table 1:** Descriptive statistics of the study major variables SD: Standard deviation, pH: Potential of Hydrogen, BMI: Body mass index, HCO3: Bicarbonate

Variables	Minimum	Maximum	M	SD	Skewness	Kurtosis
Weight	34.0	100.0	61.44	15.24	.74	-.10
Height	147.0	182.0	162.17	7.69	.25	-.27
BMI	14.50	38.60	23.16	4.6	.62	.87
Time to resolution	1	78	20.82	16.51	1.51	2.05
Admission serum PH	6.79	7.350	7.16	.142	-.90	-.09
Admission HCO3	3.0	21.9	12.15	4.66	.18	-.87
Admission sodium	117	150	133.24	5.83	.09	.50
Anion gap	-5.1	51.4	18.49	9.50	.81	3.04
Admission serum potassium	2.70	9.40	4.76	1.11	1.22	3.55
Admission serum chloride	78.0	125.0	102.69	8.56	.04	.52
Admission blood glucose	163.00	1116.00	484.07	181.99	.82	1.42
Length of stay (hours)	16	348	73.19	56.73	2.49	8.58

The results indicated that of 67 DKA patients, the majority (56.7%) were female and approximately 27% had comorbidities. Furthermore, more than 43% of patients had a precipitating factor of omission of insulin, followed by infection (12%). The results indicated that 97% of the patients improved during the study time; only 3% had Discharge against medical advice (Table 2).

**Table 2:** Demographic and clinical characteristics of the study total sample (N=67) DAMA: Discharge against medical advice

Respondents Characteristics	N	%
Gender		
Male	29	43.3
Female	38	56.7
Outcome		
Improved	65	97.0
DAMA	2	3.0
Comorbidity		
Yes	18	26.8
No	49	73.1
Precipitating Factors		
Necrotizing pancreatitis	1	1.5
Renal disease	1	1.5
Stress due to exam	1	1.5
Infection	8	11.9
Insulin omission	29	43.3
Trauma	1	1.5
N/A	13	19.4
Newly diagnosed	6	9.0
No precipitating factor	7	10.4

To assess the correlation between hospital LOS and demographic characteristics including weight, height, age, metabolic parameters (pH, HCO3, potassium, sodium, chloride, anion gap, and blood glucose), and BMI, a Pearson’s correlation coefficient test was used. The findings indicated that hospital LOS was significantly and positively associated with age (p < 0.01), weight, BMI, admission blood glucose level (p < 0.05), and admission serum potassium (p < 0.01). Longer LOS was positively associated with a higher level of admission blood glucose, greater BMI, older age, and increased weight. However, hospital LOS was significantly and negatively associated with admission serum pH (p < 0.01). The DKA patients who had high serum pH on admission had a shorter LOS. The association of hospital LOS with other admission metabolic parameters was not statistically significant (p > 0.05). Furthermore, the findings indicated that the admission pH and admission HCO3 were significantly and negatively associated with time to resolution (p < 0.05) (Table 3).

**Table 3:** Correlation among study major variables BMI: Body mass index, pH: Potential of Hydrogen, HCO3: Bicarbonate

Variables	1	2	3	4	5	6	7	8	9	10	11	12	13
Age	--												
Weight	.486^**^	--											
Height	.359^**^	.612^**^	--										
BMI	.354^**^	.914^**^	.280^*^	--									
Time to resolution	-.207	.081	-.147	.203	--								
Admission serum PH	-.164	-.191	-.104	-.208	-.267^*^	--							
Admission HCO3	-.047	-.152	-.029	-.194	-.306^*^	.879^**^	--						
Admission sodium	-.019	-.081	-.060	-.084	-.122	-.138	-.105	--					
Anion gap	.058	-.028	.086	-.086	-.014	-.313^**^	-.362^**^	.378^**^	--				
Admission serum potassium	.376^**^	.461^**^	.395^**^	.416^**^	.125	-.377^**^	-.358^**^	-.057	.195	--			
Admission serum chloride	-.043	.062	-.111	.144	.108	-.244^*^	-.228	.306^*^	-.626^**^	-.055	--		
Admission blood glucose	.269^*^	.331^**^	.242^*^	.326^**^	.035	-.271^*^	-.188	-.266^*^	.055	.522^**^	-.144	--	
Length of stay (hours)	.395^**^	.286^*^	.093	.245^*^	.117	-.364^**^	-.231	-.024	.081	.334^**^	.014	.298^*^	--

The paired-sample t-test findings indicated that the mean of metabolic parameters including pH, HCO3, sodium, chloride, and potassium significantly increased after resolution in DKA patients (p < 0.001). However, potassium, anion gap, and blood glucose significantly decreased after resolution in DKA patients (p < 0.001). The values of Cohen’s d of all metabolic parameters were > 0.50, indicating a large effect size (Table 4).

**Table 4:** Mean difference based on admission and after resolution in terms of the study's major variables pH: Potential of Hydrogen, HCO3: Bicarbonate

	Admission		Resolution				
Variable	M	SD	M	SD	p	t (66)	Cohen’s d
pH	7.16	0.14	7.33	0.03	< .001>	-9.437	-1.15
HCO3	12.15	4.66	18.29	2.26	< .001>	-11.536	-1.41
Sodium	133.24	5.83	135.84	4.38	< .001>	-3.712	-.45
Anion gap	18.49	9.50	9.80	6.52	< .001>	6.466	.79
Potassium	4.76	1.11	3.74	0.68	< .001>	7.435	.91
Chloride	102.69	8.56	107.75	6.34	< .001>	-4.403	-.54
Blood glucose	484.07	181.99	143.14	34.64	< .001>	15.340	1.87

The independent samples t-test indicated that the mean of metabolic parameters including anion gap and potassium and chloride levels was significantly higher in males compared with female DKA patients (p < 0.05). The values of Cohen’s d for anion gap and potassium and chloride levels (0.55, 0.52, and 0.49, respectively) indicated large effect sizes. Furthermore, the findings indicated that the mean of metabolic parameters including pH, HCO3, and blood glucose was higher in females compared with male DKA patients. However, this difference was not statistically significant (p > 0.05) (Table 5). 

**Table 5:** Mean difference based on gender in terms of metabolic parameters pH: Potential of Hydrogen, HCO3: Bicarbonate

	Male (n = 29)		Female (n = 38)				
Variable	M	SD	M	SD	P	t (65)	Cohen’s d
pH	7.13	0.16	7.18	0.13	.142	-1.49	-.37
HCO3	11.55	4.96	12.60	4.44	.366	-0.91	-.22
Sodium	130.07	6.29	133.37	5.54	.837	-0.21	-.05
Anion gap	21.36	9.28	16.30	9.19	.030	2.22	.55
Potassium	5.08	0.92	4.52	1.19	.040	2.09	.52
Chloride	100.35	8.93	104.47	7.92	.050	-1.99	-.49
Blood glucose	528.35	169.67	450.27	185.98	.082	1.77	.44

**Figure 3:**
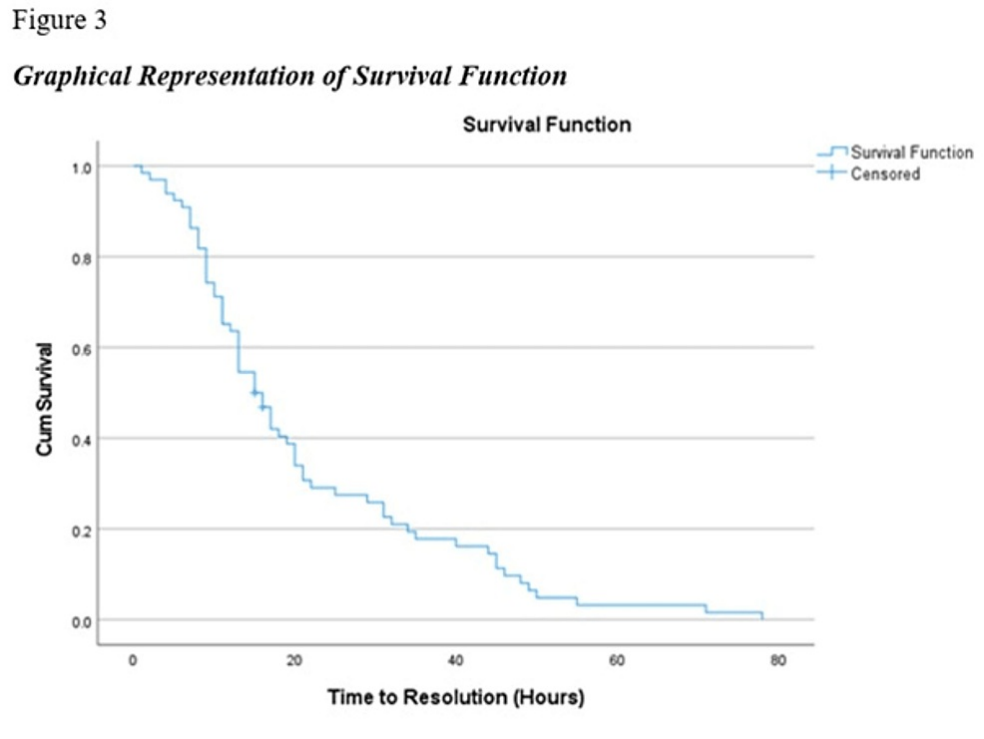
Graphical presentation of survival functions

A Kaplan-Meier estimate method was used to determine the mean and median time to resolution of DKA. The findings indicated that the mean time to resolution of DKA was 21.38 hours (17.24 hours to 25.51 hours) and the median time to resolution of DKA was 15 hours (12.21 hours to 17.79 hours) (Table 6, Figure 3).

**Table 6:** Means and medians for survival time Estimation is limited to the largest survival time if it is censored. Std.: Standard, CI: Confidence interval, LB: Lower bound, UB: Upper bound

Mean				Median			
Estimate	Std. Error	95% CI		Estimate	Std. Error	95% CI	
		LB	UB			LB	UB
21.38	2.11	17.24	25.51	15.00	1.42	12.21	17.79

A multivariate stepwise backward elimination Cox proportional hazards model indicated that of the metabolic parameters on admission, including serum pH, HCO3, sodium, anion gap, potassium, chloride, and blood glucose, only a higher serum HCO3 level (p < 0.05) was an independent predictor of longer time to resolution (Table 7). 

**Table 7:** Factors associated with slower time to resolution of DKA using a Cox proportional hazards model HCO3: Bicarbonate, HR: Hazard ratio, CI: Confidence interval

Individual Factor	HR	95% CI	p-value
Admission HCO3	1.071	1.01, 1.13	.016

A multivariate stepwise backward elimination Cox proportional hazards model indicated that of the metabolic parameters on admission, higher levels of HCO3, anion gap, potassium, and chloride and lower levels of admission serum sodium were independent predictors of minimum LOS (p < 0.05). Furthermore, the findings indicated that of the other characteristics such as age, weight, height, and BMI, only younger age was an independent predictor of minimum LOS (p < 0.001) (Table 8).

**Table 8:** Factors associated with the minimum length of stay of DKA using a Cox proportional hazards model CI: Confidence interval, pH: Potential of Hydrogen, HCO3: Bicarbonate

		95% CI		
Individual Factor	HR	Lower Bound	Upper Bound	p-value
Admission serum pH	48.189	.835	2780.341	.061
Admission HCO3	1.702	1.085	2.671	.021
Admission sodium	.580	.373	.902	.016
Anion gap	1.740	1.119	2.705	.014
Admission serum chloride	1.745	1.106	2.752	.017
Age	.950	.922	.979	< .001>

**Figure 4:**
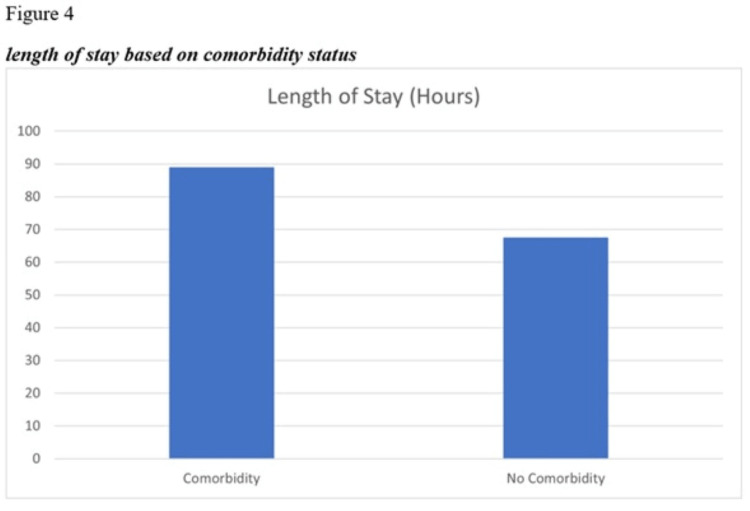
Length of stay based on comorbidity status

The independent samples t-test findings indicated that the mean LOS was higher in DKA patients who had comorbidities than those with no comorbidities. However, the difference was partially significant (p = 0.084). Furthermore, the value of Cohen’s d (0.38) indicated a medium effect size (Table 9, Figure 4).

**Table 9:** Mean difference in length of stay based on comorbidities SD: Standard deviation

	Comorbidities		No comorbidities				
Variable	Mean	SD	Mean	SD	p-value	t (66)	Cohen’s d
Length of stay	89.00	73.99	67.39	48.57	0.084	1.39	0.38

## Discussion

This study assessed the effects of different parameters influencing the time to resolution of DKA and the predictors of LOS among hospitalized patients with DKA. The results indicated that higher HCO3, potassium, and chlorine levels; younger age; and lower levels of serum sodium on admission were independent predictors of a minimum LOS. In contrast, only a high serum HCO3 level was an independent predictor of a longer time to resolution. In this study population, more females experienced DKA, and patients had an improvement rate of 97%. The mean LOS for DKA patients with a mean BMI of 23.16 was 73.19 hours. The DKA patients with higher blood glucose, higher BMI, and older age, in contrast with the DKA patients with elevated serum pH, had longer stays in the hospital. Results also indicated that the mean of most metabolic parameters increased and potassium, anion gap, and blood glucose decreased after resolution in DKA patients. Compared to female patients, male patients had a greater increase in the mean of the metabolic parameters of anion gap and potassium and chloride levels. The opposite was seen for the metabolic parameters of pH, HCO3, and blood glucose. Younger individuals were less prone to a more extended stay at the hospital.

Our study suggested that patients with DKA spent around 73.19 hours in the hospital. This result differed from the findings of previous studies. One study conducted by Mekonnen et al. found a LOS of DKA patients of 4.64 days, which is approximately 111 hours [20].

We also noticed that more females experienced DKA, as 56.7% of the patient population was female. This finding is consistent with those of previous studies. According to a systematic review, 11 studies have concluded that women were more commonly affected by DKA compared to men, regardless of age, and three studies found the opposite result [21].

The DKA patients with a high level of glucose on admission, greater BMI, older age, and increased weight stayed in the hospital for extended periods. Conversely, patients with elevated serum pH had shorter stays. Another study conducted on hospital LOS in Ethiopia found similar results [22]. In this study, the authors found that patients between 35 and 44 years of age had a shorter LOS than those between 55 and 64 years of age. The fluctuation in blood glucose levels also increased the patient's hospital stay. In addition, 83% of patients with moderate DKA (defined as DKA with high serum pH) had shorter LOS [22].

We also assessed the impact of DKA resolution on patients’ metabolic parameters. High pH, HCO3, sodium, chloride, and potassium were found after DKA resolution. In comparison, blood glucose decreased after DKA resolution. A study conducted in Ethiopia found similar results [22]. Our study used the ADA criteria for DKA resolution, which states that the pH level must be < 7.30, the HCO3 level must be < 18 mEq, and the anion gap must be < 12 [23]. According to Table 4, the mean pH is 7.36 and the HCO3 level is 18.29 at resolution. The values increased for serum pH and HCO3, with a slight difference from the standard criteria of DKA resolution. In our study, the anion gap was < 12, which is per the standards. The standard for blood glucose level in DKA resolution is < 200 mg/dL; in our study, the mean blood glucose level after the resolution was less than the standard at 141.14 mg/dL.

Our study found some differences in metabolic parameters between male and female patients. Anion gap, and potassium and chloride levels were higher in male patients, whereas female patients had higher pH, HCO3, and blood glucose levels. Our study was the first to note these differences, as no significant evidence is found in other studies. However, many studies have observed that the prevalence of DKA is higher in females than in males [21]. Another study also suggested that the length of stay for men and women remained the same [24].

In our study, the median time to DKA resolution was 15 hours, and the mean time is 21.38 hours. Another study at St Vincent's Hospital Melbourne found a median time to DKA resolution was 11 hours [14]; thus, the time to improvement was longer in our study.

A high serum HCO3 level was found to be an independent predictor of a longer time to resolution in our study. Another study on metabolic parameters and time to resolution suggested that lower admission pH and higher admission potassium levels are independent predictors of time to resolution [14]. We did not observe these results in our study.

We also found that age was the only independent predictor for a lower LOS for DKA patients among characteristics such as age, weight, height, and BMI. One study conducted in the United Kingdom also found that age is the only predictor of a lower LOS [25]. No other studies have replicated our observations that HCO3, anion gap, potassium, and chloride are independent predictors of a lower LOS.

We found that DKA patients with comorbidities had a longer hospital LOS than patients with no comorbidities. In this regard, our study provides a new angle to studies conducted on DKA patients as no such relationship can be found in other studies.

Our study proves beneficial to understanding DKA in patients. It provides a new perspective by relating DKA patients with comorbidities to a longer stay. It also refers to multiple physical parameters associated with DKA. Our study may be improved by increasing the sample size to determine if different results are found.

## Conclusions

We intended to determine the various factors affecting the resolution of metabolic parameters in DKA patients and their association with the length of stay in our patients. The study found that patients with DKA spent around 73.19 hours in the hospital, and the mean time to DKA resolution was 21.38 hours. The omission of insulin was the leading precipitating factor for DKA (43%), followed by infection (12%). This study provides insight into the different parameters affecting LOS and the time to resolution in patients with DKA, including a higher pH, HCO3, potassium, chlorine, younger age, and lower level of serum sodium on admission which were independent predictors of a minimum LOS. In contrast, only a high serum HCO3 was an independent predictor of a longer time to resolution. Our study contributes to the understanding of DKA in hospitalized patients in Saudi Arabia by identifying multiple clinical and biochemical parameters affecting their course of disease and disease outcome.
